# Revealing the Hidden Diagnostic Clues of Male Infertility from Human Seminal Plasma by Dispersive Solid Phase Extraction and MALDI-TOF MS

**DOI:** 10.3390/ijms231810786

**Published:** 2022-09-15

**Authors:** Serena Correnti, Mariaimmacolata Preianò, Pierpaolo Murfone, Annalisa Fregola, Massimo Bitonti, Rocco Savino, Rosa Terracciano

**Affiliations:** 1 Department of Health Sciences, Magna Græcia University, 88100 Catanzaro, Italy; 2 Department of Experimental and Clinical Medicine, Magna Græcia University, 88100 Catanzaro, Italy; 3 Biopepticom S.R.L., 88100 Catanzaro, Italy; 4 Department of Medical and Surgical Sciences, Magna Græcia University, 88100 Catanzaro, Italy

**Keywords:** MALDI-TOF/TOF, mass spectrometry, proteome, seminal plasma, male infertility, seminal fluid, biomarker, semenogelins, dispersive solid phase extraction

## Abstract

Seminal plasma (SP) mirrors the local pathophysiology of the male reproductive system and represents a non-invasive fluid for the study of infertility. Matrix-Assisted Laser Desorption/Ionization-Time-of-Flight Mass Spectrometry (MALDI-TOF-MS) provides a high-throughput platform to rapidly extrapolate the diagnostic profiles of information-rich patterns. In this study, dispersive solid phase extraction (*d*-SPE) combined with MALDI-TOF-MS was applied for the first time to the human SP, with the aim of revealing a diagnostic signature for male infertility. Commercially available octadecyl (C_18_)-, octyl (C_8_)-bonded silica sorbents and hexagonal mesoporous silica (HMS) were tested and the robustness of MALDI-TOF peptide profiling was evaluated. Best performances were obtained for C_18_-bonded silica with the highest detection of peaks and the lowest variation of spectral features. To assess the diagnostic potential of the method, C_18_-bonded silica *d*-SPE and MALDI-TOF-MS were used to generate enriched endogenous peptide profiles of SP from 15 fertile and 15 non-fertile donors. Principal component analysis (PCA) successfully separated fertile from non-fertile men into two different clusters. An array of seven semenogelin-derived peptides was found to distinguish the two groups, with high statistical significance. These findings, while providing a rapid and convenient route to selectively enrich native components of SP peptidome, strongly reinforce the prominent role of semenogelins in male infertility.

## 1. Introduction

Human seminal plasma (SP) is an acellular fluid, easily obtained after semen centrifugation, which contains specific proteins and endogenous peptides originating from the testis, epididymis, and male accessory glands [1,2]. Due to its intrinsic nature, being much closer to the male reproductive tract, it is significantly more enriched than serum and urine in secreted and/or shed proteins relevant for the study of infertility, male disorders, and other related pathologies [3]. SP has therefore captured a growing interest as a clinical sample for noninvasive diagnostics, with special attention being paid to the highly complex mixtures of small peptides present therein, which may offer a great potential for information rich patterns for clinical diagnosis. Indeed, several investigations have identified SP peptides involved in sperm motility and fertility [4,5]. Other studies have recognized the importance of SP peptides not only as putative markers for primary prostate cancer diagnosis [6] but also for their potential role in bactericidal activity [7,8]. Finally, recent studies have highlighted the pivotal role of specific peptides released by physiological cleavage of semen coagulum proteins (Semenogelins and Prostatic acid phosphatase) in forming amyloidogenic fragments, which are abundant in SP and which boost semen-mediated enhancement of HIV infection [9,10].

The delineation of new omics platforms, which accurately and rapidly provide a wide array of molecular entities from clinical specimens, including tissues and bodily fluids, may be of interest in the field of diagnostic and personalized medicine. With the rapid development of mass spectrometry (MS) technology, Matrix-Assisted Laser Desorption/Ionization-Time-of-Flight Mass Spectrometry (MALDI-TOF MS) has gradually increased its applications in biomedicine and clinical diagnostics, owing to its low acquisition times, ease of use, ruggedness, very high automation and throughput, and good sensitivity [11,12]. In particular, in this direction, MALDI-TOF MS provides an accessible and high-throughput platform to rapidly extrapolate by comparative analysis information-rich patterns arising from altered expression of specific peptides between normal and pathological conditions [13,14,15,16].

However, until now, in the case of human SP only a few investigations used MALDI-TOF MS either to assess fingerprints of SP lipids [17] or to assess the N-glycome profile for human SP [18]. Liu et al., by isobaric tags for relative and absolute quantitation (iTRAQ) labeling and liquid chromatography (LC)-MALDI strategy discovered infertility-related SP proteins in men with normal semen parameters and clinical pregnancy by R-ICSI after IVF failure [19]. However, MALDI-TOF peptidic profiles of non-digested human SP is reported only in one study. Fung and colleagues described direct analysis of unfractionated human SP showing peptide peaks features in a *m*/*z* range from 500 to 10,000 [20]. Additionally, only one investigation based on Surface-enhanced laser desorption/ionization (SELDI)-TOF MS reported differential protein expression in SP from fertile and infertile males [21]. In view of the above reported considerations, acquisition of rich and informative MALDI-TOF spectra preserving valuable information about endogenous and naturally occurring peptides of SP might be an attractive strategy to deliver fast and sensitive clinical diagnostic assays for pathologies and disorders of the male reproductive tract. In order to reach this goal, a practical and efficient SP peptide enrichment strategy is required before MALDI-TOF MS analysis. Currently, solid-phase extraction (SPE) prior to MALDI-TOF MS analysis is a rapid and convenient tool for profiling of clinical specimens [22]. We previously developed a procedure based on dispersive-SPE (*d*-SPE) in which the sorbent phase is suspended in a specific bio-fluid, providing more effective interaction between the sorbent and the peptides [23,24]. In the present study, for the first time, we applied a *d*-SPE coupled to MALDI-TOF MS to the enrichment and detection of human SP peptide patterns. Commercially available octadecyl (C_18_)- and octyl (C_8_)-bonded silica sorbents and hexagonal mesoporous silica (HMS) were used to enrich and selectively harvest peptidic components of SP. We systematically optimized a convenient procedure based on different sorbents performances, assessing the ability of the method to generate highly stable and reproducible spectra. Finally, as proof-of-concept a key peptide-pattern within spectra was extrapolated by differential display statistical analysis that was able to distinguish between fertile and unfertile groups.

## 2. Results and Discussion

### 2.1. Pre-Analytical Assessment of Residual Proteolytic Activity in SP

It is well accepted that the presence in SP of proteins as well as tissue-specific mediators should provide new insights in the origin of male infertility [25]. In particular, the expression in SP of naturally occurring peptides, mainly arising from proteolysis of abundant and larger proteins, might be correlated to specific function of male reproductive organs [26,27,28]. As a result, the complexity and large variety of the size and the charge of peptides make peptidomic analysis of SP quite challenging. Therefore, in this study, we firstly assessed pre-analytical and analytical variables, aiming to establish an optimized protocol based on *d*-SPE coupled to MALDI-TOF/TOF MS for peptide pattern analysis of human SP in the low molecular weight mass spectral range. It is well known, that sample collections and processing procedures may significantly affect mass spectra profile. In particular, since semen liquefaction depends on a complex proteolytic cascade [26,27,28], a major challenge with SP low molecular weight profiling study is to check and to monitor if residual proteolytic activity is still present in SP and if it might influence the spectral readouts. The addition of a protease inhibitor cocktail (PIC) containing 4-(2-aminoethyl) benzenesulfonyl fluoride hydrochloride (AEBSF), a serine protease inhibitor that blocks PSA activity, was already described by Robert et al. [28]. However, in order to set up a valid pre-analytical processing protocol for our study, the stability of SP peptide profile obtained from a normozoospermic healthy volunteer was firstly investigated. PIC containing AEBSF was immediately added after the liquefaction of coagulum as described in the Material and Methods section. Specifically, the sample was prepared in three independent experiments and the variation of the total peak numbers between samples with and without PIC was assessed at several time points, more precisely at 0 and after 60, 90, 120, and 150 min at room temperature and after 1 and 120 days of storage at −80 °C. The total number of *m*/*z* peaks with a signal-to-noise (S/N) ≥ 10 detected in the MALDI-TOF mass spectra is shown in Supplementary Figure S1 as function of the time. No statistically significant variation in peak number was observed neither up to 2.5 h at room temperature nor up to 120 days of storage at −80 °C. Moreover, no statistically significant difference in peak number was observed at any time point between samples with and without the use of PIC. Definitively, these data demonstrate that SP is stable for at least 2.5 h at room temperature and for at least 4 months when stored at −80 °C. Additionally, any potential variation that might be observed between different cohorts of subjects would hardly be an artifact due to storage conditions, provided that the analysis is performed at any time within 4 months from the collection if the sample is stored at −80 °C within 2.5 h. Therefore, it might be tempting to speculate that no significant, residual proteolytic activity is still present in SP after the liquefaction of coagulum because no statistically significant difference in peak number was observed at any time point between samples with and without the use of PIC.

### 2.2. d-SPE Enrichment with Hydrophobic C_18_ and C_8_ silica Sorbents and with HMS 

Selective enrichment strategies before MALDI-TOF MS analysis may represent an important tool to finely modulate the low molecular weight profiling of clinical samples [22,29]. In fact, depending on the efficiency of the extraction method used, poor, medium, or rich peptide fingerprints can be obtained. A molecular profile rich in peaks is highly desirable, as more informative pattern diagnostic features could be achieved for discriminant peaks analysis [15,30]. In this investigation, our efforts were mainly devoted to identifying an optimal sample preparation method suitable for desalting and efficiently enriching peptides from human SP specimens in order to generate rich informative and robust MALDI-TOF screens. 

Ideally, MALDI-TOF analysis of a clinical specimen should be accomplished by as few as possible processing steps in order to reduce sample loss. Additionally, it would be necessary to remove interferences and contaminants, which might generate high background and ambiguous peaks in the spectra. Another important requirement is to enrich the analytes of interest, in our case SP peptides, for increased sensitivity. SPE is a well known separation procedure based on the property of a chromatographic medium (the solid phase) to sequester one analyte or a subset of them from a specific solution of interest and is normally used not only for food matrices but also for clinical specimens. In *d*-SPE, the higher contact surface between the solid phase and the analytes allows reducing time needed to reach the extraction equilibrium in comparison to SPE. Consequently, *d*-SPE requires a shorter extraction time, providing a more effective capture of analytes. Owing to its outstanding rapidity, to low solvent consumption compared to standard SPE, to high efficiency, and to its wide applicability, this procedure is attracting growing interest [24,31,32].

Based on this rationale, in this study we explored for the first time commercially available C_8_- and C_18_-bonded silica beads and HMS as harvesting sorbents for human SP peptide enrichment by *d*-SPE. Optimal conditions were assessed for adsorption, washing, and elution steps, in order to ensure the best MALDI-TOF mass spectra in terms of number of detected peaks, repeatability on peak heights, area, and signal-to-noise ratio (see Supplementary Materials and Supplementary Tables S1–S4, Supplementary Figures S2–S5). Other critical parameters, such as the number of absorbents, the pH, the type of washing solvent, and the elution time, were established on the basis of our previous results obtained with clinical biological matrices for the enrichment/extraction of peptides [24].

**Figure 1:**
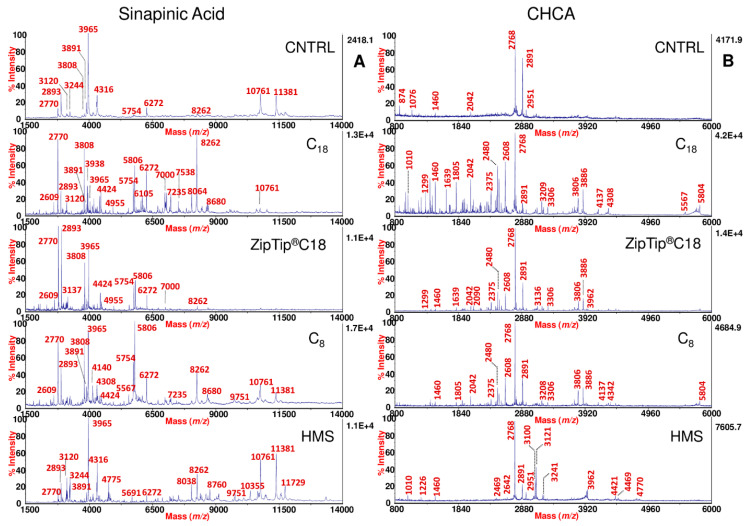
MALDI-TOF MS full view of SP using SA and CHCA matrices. MALDI-TOF mass spectra of SP obtained from the same fertile normozoospermic man using SA (**A**) and CHCA (**B**) as matrix, before and after processing by C_18_, ZipTip^®^C18, C_8_, and HMS. For SA, the spectra are shown in the *m*/*z* range from 1500 to 14,000. For CHCA, the spectra are shown in the *m*/*z* range from 800 to 6000, with labeled monoisotopic peaks.

**Figure 2:**
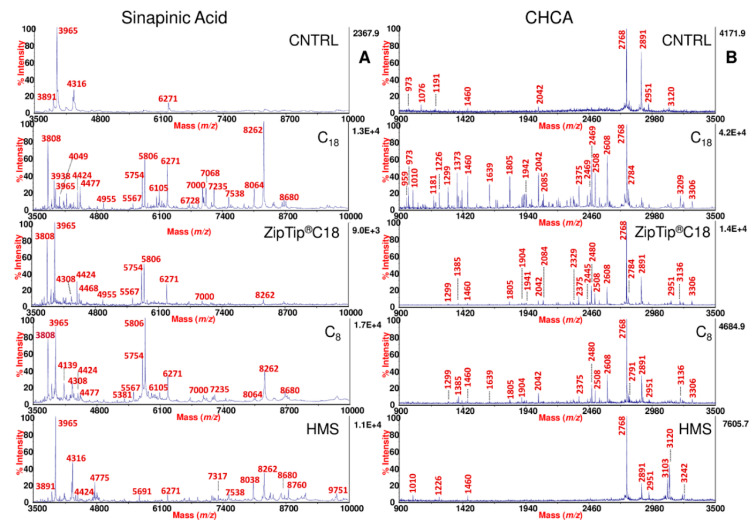
MALDI-TOF MS zoom view of SP using SA and CHCA matrices. MALDI-TOF mass spectra of SP obtained from one fertile normozoospermic subject using SA (**A**) and CHCA (**B**), before and after processing by C_18_, ZipTip^®^C18, C_8_, and HMS. For SA, the spectra are shown in the *m*/*z* range from 3500 to 10,000. For CHCA, the spectra are shown in the *m*/*z* range from 900 to 3500, with labeled monoisotopic peaks.

Silica bonded C_18_ and C_8_ sorbents were tested owing to their well-known interactions for hydrophobic peptides. In general, slight differences in selectivity are observed between C_18_ and C_8_ sorbents [33]. Instead, in the case of HMS a larger spectrum of peptides might be extracted because the textural properties of these silica sorbents coupled to the characteristic wormhole mesostructured ensure very satisfactory results for integrating size selectivity with effective adsorptive mechanism [24]. Representative examples of the SP peptidic profiles obtained from the same sample before and after *d*-SPE processing with the different sorbents used in our study are shown in Figure 1 and Figure 2. 

In particular, the spectra were acquired both in sinapinic acid (SA) and α-cyano-4-hydroxycynnamic acid (CHCA), which are the most common MALDI matrices used for peptide profiling. Different *m*/*z* ranges are shown, in order to better highlight both in “full” (Figure 1) and “zoom” (Figure 2) views, the high peaks enrichment provided by the use of the *d*-SPE processing.

**Figure 3:**
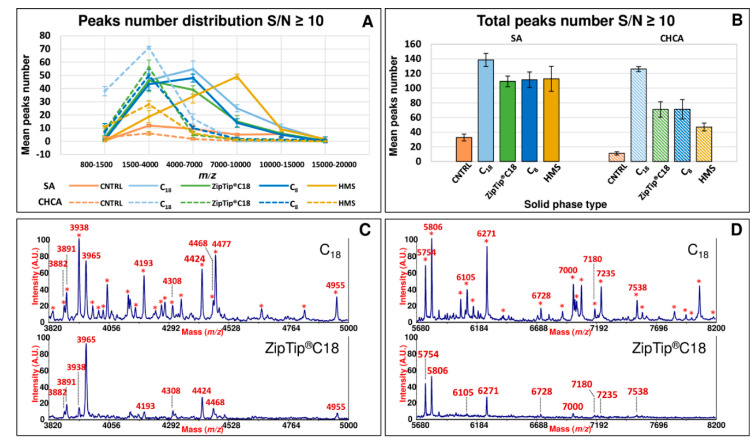
MALDI-TOF peaks number distribution using different *d*-SPE sorbents and peptides enrichment comparison between C_18_ *d*-SPE and ZipTip^®^C18. Panel (**A**) shows the mean peaks number distribution for SP samples over different *m*/*z* ranges using SA and CHCA before and after processing by C_18_, ZipTip^®^C18, C_8_ and HMS. Panel (**B**) displays the total peaks number detected for SP samples using SA and CHCA before and after processing by C_18_, ZipTip^®^C18, C_8_, and HMS. Panels (**C**,**D**) display the comparison of spectral portraits in SA of SP between C_18_ and ZipTip^®^C18 in the *m*/*z* range from 3820 to 5000 and from 5680 to 8200, respectively. Asterisks indicate peaks selectively extracted by C_18_ sorbent, which are absent or detected with a lower signal intensity in ZipTip^®^C18 treated sample.

Commercial ZipTip Pipette Tips equipped with C_18_ chromatographic media (conventionally used SPE approach) were also included in this study for comparative purposes. As expected, the use of these pre-treatment methods, removing salts, interferences and other contaminants, which generate signal suppression in the MALDI-TOF analysis, significantly amplified the peptidic repertoire of SP compared to the same untreated sample (Figure 1 and Figure 2). Comparing each of the spectral view of *d*-SPE processed samples (Figure 1 and Figure 2) to the same untreated sample, the fine tuning in the peptide profiling through the different sorbents allowed the detection of specific subset of SP peptides, which were missing without pre-treatment. Moreover, in the described dispersive procedure (see Section 3), since the contact area between the sorbents and the analytes is amplified in comparison to the classical SPE, a more effective interaction reduces the times of the protocol and the volumes of the solvent required for the elution of the peptides retained on the sorbents. In fact, only 10/15 min are required for the adsorption step and few microliters (15/25) for the elution of peptides adsorbed on the sorbents (Supplementary Table S1–S3). Dispersive methods appear to generate MALDI-TOF spectral portraits with an increased number of peaks and with higher intensity, area, and S/N after the enrichment (see Figure 1 and Figure 2 and also Figure 3A,B). Generally, for all sorbent tested, in the case of SA, the best detection of the SP peptide peaks was observed in a *m*/*z* range from 1500 to 14,000 (Figure 1A). For CHCA, the *m*/*z* range showing the best detection was lower than that observed for SA, displaying best peaks fingerprints in the range between 800 and 6000 (Figure 1B).

A more detailed study of *d*-SPE performances was obtained through the distribution of peaks eluted by the diverse sorbents in different *m*/*z* regions of the spectrum (Figure 3A). An increase of the peak number was observed from 800 to 4000 in CHCA for all kinds of sorbents. Among all stationary phases, pre-processing using C_18_ silica sorbent showed the best performance (in term of extracted peaks) in the low mass range (1500–4000) for spectra acquired in CHCA and in the medium mass range (4000–7000) for spectra acquired in SA (Figure 3A). C_8_ silica sorbent (and ZipTip) showed a peak distributions similar to C_18_ (Figure 3A). Specifically, C_8_ sorbent paralleled the C_18_ peak distribution behavior although running in a slightly lower region of the y-axis. The similar trend in peak distribution is explained by the quite similar adsorption behavior based on hydrophobic interactions. A different peaks distribution of the eluted peaks was observed when HMS particles were used, in particular for the spectra acquired in SA. In fact, as shown in Figure 3A, the maximum of the distribution for HMS was shifted to 7000–10,000 *m*/*z* region of mass spectra acquired in SA. While, for the spectra acquired in CHCA, the maximum was observed in the same range (1500–4000) of the other sorbents. The HMS shows hydrophilic silica features and mesoporous wormhole structure that warrants not only an exceptional dispersibility but also an optimal enrichment ability for peptides based on a cut-off mechanism (depending on the mesopore diameter) and a variety of interactions [24]. Therefore, it is reasonable to observe a different shape in the distribution of peaks in the different regions of the mass spectra. The distribution of peptides for HMS mirrors mainly the size exclusion effect and, as a consequence, the majority of peaks were detected in the *m*/*z* range between 7000 and 10,000 (Figure 3A), while a low efficiency was observed for higher molecular weight species.

In the case of the CHCA, the shorter *m*/*z* ranges analyzed do not allow highlighting differences in the maximum, although a different slope is observed from 1500 to 4000. Considering the total number of peaks detected with an S/N ≥ 10, best results were obtained for C_18_ sorbent, with ~140 peaks detected over the entire *m*/*z* range from 800 to 20,000 when the spectra were acquired in SA, and ~120 peaks when the spectra were acquired in CHCA (Figure 3B). Although the total peak number was slightly lower, also C_8_ and HMS showed an optimal efficiency in SP peptide capture as illustrated in Figure 3B with comparable total peaks detected (approximately 110) in SA. In the C_18_ sorbent, the presence of a great coverage of octadecyl chain bound to the silica surface, associated to the great methylene selectivity, while reducing silanols interactions, allows for very hydrophobic interactions between the SP peptides and the stationary phase. For the C_8_, the shorter octyl chain provides less hydrophobic interactions, and maybe this provides a possible explanation for the slightly lower number of peaks extracted for this sorbent in comparison to C_18_ (Figure 3B). A lower number of peaks was detected for HMS in CHCA in comparison to the other sorbents (Figure 3B). As already stated above, the adsorptive mechanism for this mesoporous sorbent is quite different from the C_8_ and C_18_; moreover, we do not exclude that during our *d*-SPE procedure with HMS some low mass proteins might have been removed during the washing step. Furthermore, it might be also possible that a part of the low molecular weight SP peptides are bound to larger SP proteins as it happens in the case of plasma or serum peptides for the presence of carrier proteins, such as human serum albumin [34]. In this case, due to the cutoff mechanism of HMS, the large carrier proteins with bound LMWP cannot be adsorbed into the nanometric porous network wormhole channels of the HMS. This phenomenon might explain the lower number of peptides extracted by HMS in the low molecular *m*/*z* range in comparison to the other sorbents (Figure 3A,B). 

Figure 3C,D show the comparison of spectral portraits in absolute intensity units of SP between C_18_ and Ziptip C_18_ in the *m*/*z* range from 3820 to 5000 and 5680 to 8200, respectively. In particular, several peaks (indicated by asterisks) extracted by C_18_ sorbent are not observed or show a lower intensity in the case of ZipTip C18. These data highlight the higher performance of *d*-SPE in comparison to SPE.

Although inter-individual variations of protein composition in SP might influence differential proteomic/peptidomics analysis [35,36], our effort was to standardize and optimize the experimental procedure with the aim to provide a robust tool for biomarker discovery. In order to warrant a robust analytical tool, repeatability and reproducibility of the method were assessed for spot-to-spot and within-spot reproducibility as reported in the Materials and Method section and in the Supplementary Materials. The results are summarized in Supplementary Figures S2–S5. Mean CVs% obtained for peak heights, area and S/N ranged between 10% and 15% both for inter and intra spot for all spectra obtained after pre-treatment, while in the case of the untreated sample, the CV% was comprised between 20% and 25% (Supplementary Figure S5). Among all *d*-SPE sorbents and ZipTip, the lowest variation in signal intensity, area, and S/N was observed for C_18_ *d*-SPE (mean CVs less than 10%-see also Supplementary Figure S5). Overall, the data showed a well adequate analytical robustness, suggesting that these SP peptidic fingerprints might be part of a diagnostic profile for properly weighing substantial alterations between fertile and infertile groups, likely reflecting the physiological or pathological state of the male reproductive tract.

### 2.3. Differential Comparative Analysis between Fertile and Infertile Groups

To assess the diagnostic potential of this platform, 15 SP specimens from normozoospermic fertile subjects and 15 from non-fertile subjects were analyzed and peptide enriched fingerprints by C_18_ *d*-SPE were compared by statistical data analysis and statistical assessment model (principal component analysis-PCA). Table 1 reports the clinical characteristics of the cohort subjects enrolled in this study including all semen parameters for semen subtype classification according to the World Health Organization (WHO) 2010 guidelines [37]. Further details of the study groups are listed in Supplementary Table S5.

**Figure 4:**
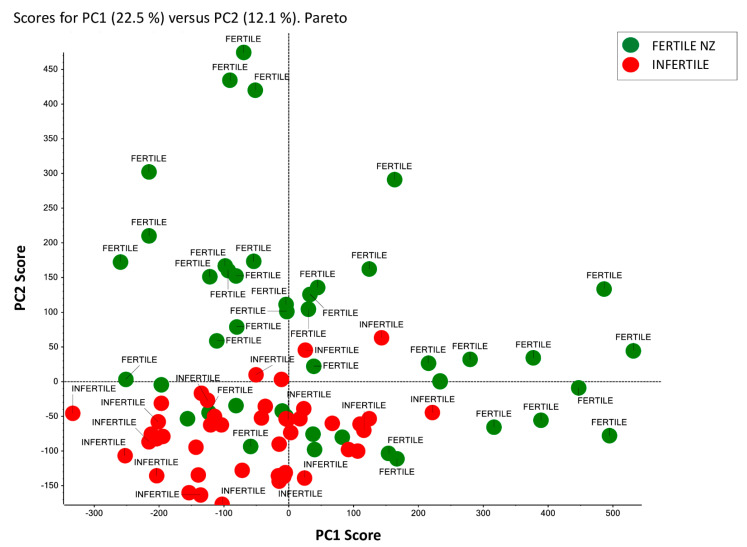
Unsupervised PCA of SP from fertile and infertile men enrolled in this study. Unsupervised PCA scores plot acquired by MarkerView™ software represents SP clustering of fertile (green dots) vs. infertile men (red dots). Pareto scaling was applied on the MALDI-MS data set.

As an explorative effort, we assessed by PCA if SP C_18_ sorbent enriched peptide MALDI signatures from the 30 donors (15 fertile vs. 15 infertile) allow any possible clustering in an unsupervised modality. PCA is a processing tool that analyzes the variance of a dataset in an unsupervised manner, considering the expected and unexpected variance, with high dimensionality data sets [38]. In particular, PCA, performed with the use of MarkerView™ software, reduces the complexity of a mass spectral dataset. Based on an unbiased assessment of inherent spectral differences, this tool shows any intrinsic clustering of examined samples. The results are shown in Figure 4: two separate clusters were visualized indicating that unsupervised PCA satisfactorily segregates infertile patients from fertile normozoospermic controls. Infertile individuals (red dots) appear quite tightly clustered and quite cleanly separated in the plot from the fertile individuals (green dots). Samples clustering and differentiation confirm data homogeneity (Figure 4) and strongly suggest that the platform described might provide a useful baseline resource for future fertility biomarker studies. 

Table 2 lists the peaks differentially expressed between the fertile and infertile groups in a statistically significant manner. 

**Figure 5:**
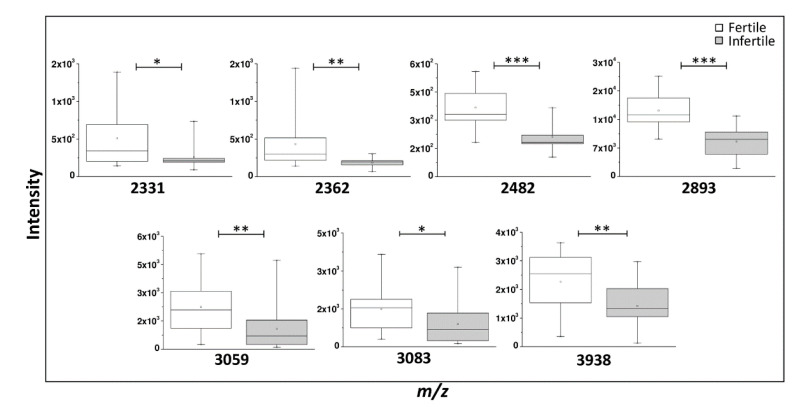
Main discriminant peaks box plot analysis. Box plot of the peak intensities for statistically discriminant *m*/*z* signals between 15 fertile and 15 infertile men performed by OriginLab^®^ software. The *p* values were calculated with unpaired *t*-test on normalized peak intensity and the asterisks show the level of significance between the two groups; * *p* values < 0.01, ** *p* values < 0.005, *** *p* values < 0.0001.

The graphical comparison of the normalized peak heights between the two groups, illustrated in the box plots reported in Figure 5, shows that each main discriminant *m*/*z* peak is downregulated in infertile men with very significant p-value for the peaks 2893 (*p* = 0.0000006) and 2482 (*p* = 0.00003). Highly significant *p*-values were also found for the peaks 2362, 3059, and 3938 with *p*-values < 0.005. The other peaks 2331 and 3083 show a *p*-value ≤ 0.01 (Figure 5).

**Figure 6:**
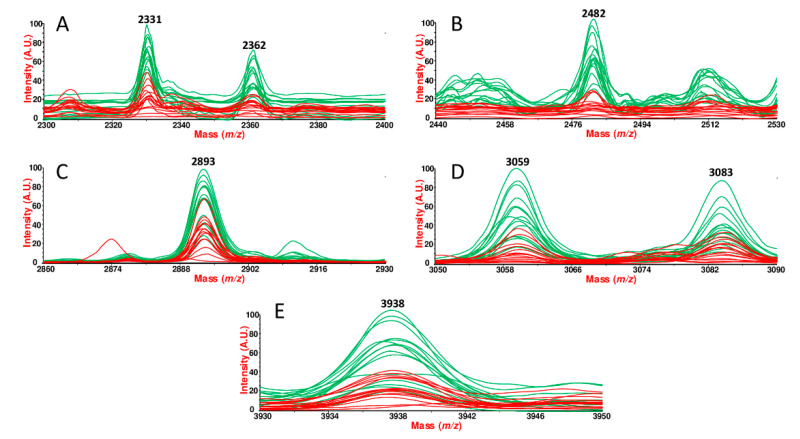
Traces overlay for discriminant peaks between fertile and infertile men. MALDI-TOF fingerprints comparison of SP samples between fertile (green) subjects and infertile (red) patients, obtained using Data Explorer software. Spectra overlay in absolute units highlights the peak intensity variation resulted statistically significant for the *m*/*z* peaks 2331 and 2362 (**A**), 2482 (**B**), 2893 (**C**), 3059 and 3083 (**D**), 3938 (**E**).

In order to better mark differential peak expressions, the overlaid mass traces from the 15 fertile (green profiles) and 15 infertile men (red profiles) are shown in the *m*/*z* regions from 2300 to 2400 (Figure 6A) for the peaks 2331 and 2362. The other *m*/*z* peaks are shown in the spectral ranges from 2440 to 2530 (Figure 6B), from 2860 to 2930 (Figure 6C), from 3050 to 3090 (Figure 6D), and in the *m*/*z* range from 3930 to 3950 (Figure 6E).

The seven differentially expressed peptides were then identified by direct sequencing by MALDI-TOF/TOF mass analysis, as shown in Table 2 and Supplementary Figure S6 in which the primary sequences, the identities, and the MS/MS mass spectra are shown. These peaks resulted as array of seven Semenogelins-derived peptides. In particular, four peaks (*m*/*z* = 2331, 2482, 2893, 3059) originated from Semenogelin-1 (SEM I) and three peaks (*m*/*z* = 2362, 3083, 3938) were fragments semenogelin-2 (SEM II). Interestingly, the SEM I fragments with *m*/*z* 2362 and 3059 (see Table 2) shared a common aminoacidic sequence 248–267. More intriguingly, the SEM II fragment with *m*/*z* = 3083 covers the sequence 248–273, which shows very high sequence similarity with SEM I fragment 248–273.

Semenogelins are abundant proteins in SP; as a consequence, the amount of these fragments may reflect the balance between the activity of specific enzymes (including proteases) and their inhibitors [6,39]. In fact, immediately after ejaculation, the spermatozoa are immobilized in the semen coagulum matrix. The semenogelins, which are the main proteins forming the semen coagulum, are proteolytically cleaved by prostate-specific antigen (PSA also known as KLK3). Owing to its proteolytic activity, PSA breaks up the coagulum bound to the spermatozoa surface, which increases their motility [40]. The resulting liquefaction of the clot after semenogelins fragmentation, warrants the physiological motility required by spermatozoa to reach the female reproductive tract [40,41]. Interestingly, PSA expression is decreased in infertile subjects with reduced sperm motility [42,43]. Moreover, in a recent study, comparative and quantitative proteome analysis between two groups of men with oligoasthenozoospermia (total number of spermatozoa and percentages of progressively motile spermatozoa below the lower reference limits) and normozoospermia (total number of spermatozoa and percentages of progressively motile and morphologically normal spermatozoa are equal to or above the lower reference limits), PSA was found decreased in SP of men with oligoasthenozoospermia [44]. Therefore, it is tempting to speculate that the decreased level of expression of semenogelins-derived peptides in the SP of infertile men may be associated to a decreased expression of PSA in the unfertile group. 

SEM I was found increased in oligoteratozoospermia (total number of spermatozoa and percentages of morphologically normal spermatozoa are below the lower reference limits) [45], while in the recent proteomics study by Martins et al. [46] both SEM I and SEM II resulted under-expressed in primary and secondary infertile individuals in comparison to the control group. However, western blot validation experiments confirmed only SEM II decreased in primary infertility, while any change in the expression of SEM I and SEM II by western blot analysis was observed in secondary infertility group [46]. 

These contrasting findings on semenogelins change of expression may arise from the intrinsic limitations of bottom up approaches and the presence in SP of both intact semenogelins and peptide-derived semenogelins. In fact, in a bottom up approach all the proteins are digested before LC-MS/MS analysis, therefore it is not possible to discriminate if the peptides that contribute to the identification of SEM I and II originate from intact precursors or from a fragment derived peptide. It is important to underline that, as top-down strategy, our method does not require the use of trypsin or more in general proteolytic digestion in comparison to bottom-up strategy. Therefore, the spectral readouts acquired by this platform reveal SP peptides in their native and biologically active forms.

Currently, mechanism underlying the physiological roles of the semenogelins, including their proteolytically-derived peptides, are still unclear; however, their implication and clinical relevance in male infertility is well described in literature [47,48,49]. Considering the important role of semenogelins in coagulation and liquefaction process and their great impact on fertility, our platform provides a new rapid and robust tool to reveal new potential diagnostic semenogelin-derived signature of male infertility hidden in SP. The data obtained in this pilot study will be further extended to a larger cohort in order to validate these preliminary findings. Additionally, the use of other sorbents, such as C4 and mesoporous silica, are currently under investigation in our group with the aim to test their potential to amplify the peptidomic repertoire of SP.

## 3. Materials and Methods

### 3.1. dSPE Sorbents

Silica gel C_8_-Reversed phase (60759) and Discovery^®^ DSC-18 SPE Bulk Packing (52600-U) were purchased from Supelco (Merck, Darmstadt, Germany). 

HMS (code 541036 wormhole silica mesostructured) was purchased from Sigma-Aldrich (St. Louis, MO, USA). HMS (code 541036 wormhole silica mesostructured). ZipTip^®^C18 was purchased from Merk Millipore Corporation (Darmstadt, Germany).

### 3.2. Reagents

All the chemicals and reagents were analytical grade. Water was purchased from Sigma Aldrich. Acetonitrile (ACN) (HPLC grade) and trifluoroacetic acid (TFA) (ACS grade) were obtained from Merck (Darmstadt, Germany). The MALDI matrices Sinapinic Acid (SA) and alpha-cyano-4-hydroxycinnamic acid (CHCA) were purchased from Fluka (St. Louis, MO, USA). Peptide mass standards kit for calibration of AB SCIEX MALDI-TOFTM instruments (AB Sciex, Framingham, MA, USA). Protease inhibitor cocktail (PIC; P8340, Sigma, St. Louis, MO, USA) and Pierce™ BCA Protein Assay Kit (23225, Thermo Scientific™, Rockford, IL, USA) were used, according to the manufacturer’s instructions. Ammonium bicarbonate (09830) was purchased from Sigma Aldrich (St. Louis, MO, USA).

### 3.3. Patient Recruitment

The study on the volunteer subjects was conducted in accordance with the Declaration of Helsinki and was approved by the Ethics Committee of MAGNA GRAECIA UNIVERSITY and MATER DOMINI HOSPITAL (protocol code 2014.39, date of approval 16 April 2014). All subjects were enrolled after the informed written consent was obtained from each participant. Only the first sperm evaluation was used in this analysis. Samples from patients who had vasectomy or history of orchitis, testicular trauma, sexually transmitted disease, varicocele, inguinal hernia operation, and cryptorchism were excluded. A questionnaire was distributed to obtain information on age, smoking habits, alcohol use (regular, irregular, or total abstinence), and use or abuse of other substances and drugs (yes or no). Semen from fertile men and infertile patients was obtained by masturbation into sterile containers after (3–5) days of sexual abstinence. All specimens were processed and analyzed anonymously.

The detailed clinical characteristics of the subjects enrolled in this study are summarized in Table 1.

### 3.4. Preparation of Seminal Plasma

Each collected ejaculated sample was allowed to liquefy for 15–30 min at 37 °C. Semen parameters were assessed according to the World Health Organization guidelines 2010 [37]. After the complete liquefaction of coagulum, liquefied sample from each donor, was divided into two aliquots. In one aliquot a protease inhibitor cocktail (PIC) was immediately added in a 1:100 *v*/*v* ratio. Then, both aliquots were processed to obtain SP. In particular, each clinical sample was centrifuged at 15,000× *g* for 15 min at 4 °C. The supernatant (the SP) resulted as a clear and fluid phase that was separated from pellets (debris) and cellular components, aliquoted, and stored at −80° C until use. Protein concentration of SP was determined by the bicinchoninic acid (BCA) assay according to the manufacturer’s instructions. Supplementary Table S5 summarizes for each SP sample the determined total protein content.

### 3.5. Assessment of Protease Activity in SP 

To assess stability of SP peptidic profile the freshly obtained semen from a normozoospermic fertile donor was used. After the complete liquefaction of coagulum, liquefied sample was split in two and PIC was added to one of the samples. Then, both samples were processed to obtain SP. In particular, each clinical sample was centrifuged at 15,000× *g* for 15 min at 4 °C. The supernatant (the SP) resulted as a clear and fluid phase that was separated from pellets (debris) and cellular components. Aliquots were analyzed immediately and after 60, 90, 120, and 150 min at room temperature; then, aliquots were frozen at −80°C and analyzed after 1 and 120 days of storage. The number of peaks were comparatively analyzed (Supplementary Figure S1).

### 3.6. Seminal Plasma Samples Normalization

The total protein content of SP specimens obtained from each study participant was determined by BCA assay (Supplementary Table S5). The concentrations of SP specimens collected from each donor patient, ranged from 30.7 to 82.0 mg/mL. Therefore, an adequate volume of each clinical sample was either concentrated (by vacuum centrifugation) or diluted (by the addition of deionized water) to 50 µL, in order to obtain a final concentration of 50 mg/mL and a total protein content of 2.5 mg

### 3.7. C_18_ and C_8_ d-SPE Optimized Procedure

A total of 10 mg of C_18_ or C_8_ silica sorbents were mixed with 100 µL of SP sample (50 μL of normalized SP sample in 50 μL of deionized water). The slurry was gently vortexed at room temperature for 10 min, then it was centrifuged at 2000× *g* for *2* min. The sorbent was then separated from the supernatant and was washed twice with 20 µL of 0.1% TFA. After the last wash, peptides bound to solid phase were eluted with 15 µL of a 1:1 (*v*/*v*) solution of ACN/0.1% TFA. Eluates were in part aliquoted and stored at −80 °C and, in part, immediately used for MALDI-TOF MS analysis.

Detailed experimental procedures for optimization of final protocols are described in the Supplementary Materials.

### 3.8. d-SPE HMS Optimized Procedure

A total of 10 mg of HMS were mixed with 100 μL of SP sample (50 μL of normalized SP sample in 50 μL of deionized water). The slurry was shaken at room temperature for 15 min. The suspension was centrifuged at 2000 *g* for 2 min, then HMS particles were separated from the supernatant and washed twice with 20 μL 0.1% TFA. After the last wash, species adsorbed on HMS were extracted with 25 μL of a 1:1 (*v*/*v*) solution of ACN/0.1% TFA. Eluates were in part aliquoted and stored at −80 °C and in part immediately used for MALDI-TOF MS analysis.

Detailed experimental procedures for optimization of final protocols are described in the Supplementary Materials.

### 3.9. ZipTip C18

After protein quantification, each SP sample was diluted (by the addition of deionized water) to a final concentration of 0.4 mg/mL, as suggested by the manufacturer’s instructions of ZipTip^®^C18 pipette tips.

### 3.10. MALDI-TOF MS Analysis

For MALDI-TOF MS sample preparation, a saturated matrix solution of SA in 35% ACN/0.1% TFA was prepared. The CHCA matrix was prepared by dissolving 4 mg in 1 mL of a solution prepared with 50% of ACN in 0.1% TFA. The solutions were then sonicated for 1 min. SP samples were prepared by a dry-droplet method. A total of 1 µL of non-treated SP or previously treated with HMS, C_18_, C_8_ sorbent, or ZipTip^®^C18 was mixed with 4 µL of SA or CHCA solution prepared as described above and 1 µL of the resulting mixture was spotted on the MALDI target plate (Opti-TOF 384-Well Insert, ABSciex, Framingham, MA, USA).

MALDI-TOF mass spectra were acquired on AB SCIEX MALDI-TOF/TOF 5800 mass spectrometer (ABSciex, Framingham, MA, USA), equipped with a diode-pumped, Nd:YLF laser with λ = 345 nm wavelength. Each sample was run in triplicate. For MALDI MS measurements in SA the following settings were applied: bin size was set at 4 ns, final detector voltage was 2.070 kV with multiplier value at 0.75; 3000 laser shots were accumulated for each spectrum. MS data were calibrated via external calibration using the 5800 Mass Standards kit (AB SCIEX, Framingham, MA, USA) containing insulin bovine (MH^+^ 5734.59), thioredoxin (MH^+^ 11,674.48), and horse apomyoglobin (MH^+^ 16,952.56). For MALDI MS measurements in CHCA the following settings were applied: bin size was set at 1 ns, final detector voltage was 1.980 kV with multiplier value at 0.66; 3000 laser shots were accumulated for each spectrum. MS data were calibrated via external calibration using the 5800 Mass Standards kit (AB SCIEX, Framingham, MA, USA) containing des-Arg^1^-Bradykinin (MH^+^ 904.4681), Angiotensin I (MH^+^ 1296.6853), Glu-Fibrinopeptide B (MH^+^ 1570.6774), ACTH (clip 1–17) (MH^+^ 2093.0867), ACTH (clip 18–39) (MH^+^ 2465.1989), ACTH (clip 7–38) (MH^+^ 3657.9294).

Data Explorer version 4.11 software (AB SCIEX, Framingham, MA, USA) was used for data acquisition and data processing.

### 3.11. Repeatability Assessment

The SP sample from one normozoospermic donor was used to assess the repeatability of the MALDI analysis by three independent experiments performed on non-treated (control) and treated (C_18_, C_8_, HMS,) samples. For each experiment, 6 spectra were acquired with a total of 18 replicates. 

### 3.12. Spot-to-Spot and within Spot Reproducibility 

A total of 10 samples were prepared for MALDI-TOF MS analysis. Two samples included control SP samples in CHCA and SA collected from one normozoospermic donor. The other eight samples were obtained from four different SP enrichment preparations, three using *d*-SPE with three different sorbents (C_8_, C_18_ and HMS), one using SPE by ZipTip collected from the same normozoospermic donor using control SP sample. 

For each of the ten MALDI samples three spots were loaded on the MALDI target plate. Six MALDI-TOF mass spectra were acquired from each spot. Eighteen spectra for each sample were acquired for intra and inter spot MALDI-TOF reproducibility assessment. A total of 180 mass spectra were acquired for experiments in SA. At the same manner, a total of 180 mass spectra were acquired for experiments in CHCA. S/N, peak intensity and peak area from 30 selected peaks both in CHCA and in SA acquired spectra were used for calculating spot-to-spot and within spot reproducibility (Supplementary Figure S5).

### 3.13. Differential Statistical Peptide Pattern Analysis 

MALDI-TOF mass spectra were first acquired and processed using Data Explorer version 4.11 software (AB SCIEX, Framingham, MA, USA) and subsequently analyzed for differential peptide patterns. Mass t2d data files were uploaded into MarkerView™ software 1.2.1.1 (AB Sciex, Foster City, CA, USA) and differential peptide profiling was assessed by unpaired, two-tailed Student’s t-test with aligned MALDI-TOF mass spectra and the normalized peak height for each *m*/*z* value. The list of differentially expressed peaks was then filtered by manual inspection and each peak was verified as described in a previous report [50]. Box plot analysis between fertile and infertile men for statistically discriminant *m*/*z* signals was performed by OriginLab^®^ software (version 7.0, OriginLab Corporation, Northampton, MA, USA). 

### 3.14. MALDI-TOF/TOF Sequencing Experiments

The differentially expressed peptides were directly subjected to the MALDI-TOF/TOF analysis for acquiring sequence information (Supplementary Figure S6). For the MS/MS measurements, 1 μL of SP sample was mixed with 4 μL of matrix solution (4 mg/mL of CHCA in 50% ACN and 0.1% TFA), and 1 μL of the obtained solution was spotted on the MALDI target plate. The voltage settings were 8.0 kV and 15.0 kV for the ion source 1 and source 2, respectively. Air was used as the collision gas and MS/MS spectra were acquired by accumulating twenty spectra (1000 shots each) at 1000 Hz pulse rate and laser energy setting of 5000–6000. The experimental collision-induced dissociation (CID)-MS/MS ion spectra were compared to theoretical MS/MS spectra generated from Protein Prospector (http://prospector.ucsf.edu/, accessed on 18 July 2022). In Supplementary Figure S6, the MALDI-TOF/TOF mass spectra with b and y ion series and the assigned peptide sequences of identified species are reported.

### 3.15. PCA 

MS data were exported from the 5800 MALDI ABScieX as t2d files and were then processed with MarkerView™ software 1.2.1.1 (AB Sciex, Foster City, CA, USA), with well-defined mass tolerance limits imposed for PCA. Specifically, unsupervised PCA was performed in order to visualize samples clustering. PCA results are commonly plotted in two- or three-dimensional plots that reflect the behavior of the samples (scores plot) or variables (loadings plot). Pareto scaling and no weighting was applied on the MALDI-MS data set comprising the normalized *m*/*z* peak intensities from SP peptides enriched with C_18_ sorbent using three replicate spectra for each subject. In brief, Pareto data set processing performs mean centering and scaling using square root of standard deviation of peak intensities.

## Figures and Tables

**Table 1:** Clinical characteristics of the subjects enrolled in the study.

Patient Features (Mean ± SD)	NZ ^1^ n = 15	AT ^2^ n = 2	OAT ^3^ n = 5	TZ ^4^ n = 6	OZ ^5^ n = 2
Age	28.7 ± 7.3	43 ± 2.8	31.2 ± 5.6	30.8 ± 7.9	33 ± 14.1
Ejaculated volume (mL)	3.3 ± 1.6	3.4 ± 1.3	2.3 ± 0.8	4.3 ± 2.1	3.15 ± 0.2
pH	7.7 ± 0.3	7.5 ± 0	7.7 ± 0.4	7.5 ± 0.4	8.25 ± 0.2
Sperm count (million)	209.2 ± 125.7	155.9 ± 62.3	3.4 ± 2.4	269.1 ± 72.9	20 ± 5.3
Progressive motility (%)	49.3 ± 7.3	23 ± 7.1	10.6 ± 12.3	41.2 ± 6.5	40.5 ± 7.8
Total motility (%)	61.6 ± 7	45 ± 4.2	16.8 ± 10.1	57.2 ± 8.5	53.5 ± 2.1
Normal morphology (%)	7.4 ± 3.4	2.5 ± 0.7	0.7 ± 0.5	2.4 ± 0.9	4.25 ± 0.3

^1.^ Normozoospermic subject, fertile man. In this category all semen parameters are normal within the acceptable reference values provided by the World Health Organization (WHO) 2010 guidelines: total number of spermatozoa, and percentages of progressively motile and morphologically normal spermatozoa, equal to or above the lower reference limits (total sperm number ≥ 39 × 10^6^ spermatozoa per ejaculate, sperm progressive motility ≥ 32% and normal sperm morphology ≥ 4%). ^2.^ Asthenoteratozoospermic patient, an infertile man with percentages of both progressively motile and morphologically normal spermatozoa below the lower reference limits. ^3.^ Oligoasthenoteratozoospermic patient, an infertile man with total number of spermatozoa and percentages of both progressively motile and morphologically normal spermatozoa below the lower reference limits. ^4.^ Teratozoospermic patient, an infertile man with percentage of morphologically normal spermatozoa below the lower reference limit. ^5.^ Oligozoospermic patient, an infertile man with total number of spermatozoa below the lower reference limit.

**Table 2:** Discriminant peaks significantly different between fertile (n. 15) and infertile (n. 15) men.

*m*/*z* ^1^	Uniprot ID (Accession Number)	Protein Identity	Peptide Sequence	Study Groups and Peptide Expression Level	*p* Value ^2^
2331	P04279	SEM I; Fragm:330–349	ITIPSQEQEHSQKANKISYQ	↑Fertile/↓Infertile	0.007
2362	Q02383	SEM II; Fragm:248–267	HGPKDIFTTQDELLVYNKNQ	↑Fertile/↓Infertile	0.001
2482	P04279	SEM I; Fragm:195–215	VLQTEELVANKQQRETKNSHQ	↑Fertile/↓Infertile	0.00003
2893	P04279	SEM I; Fragm:428–453	HGSHGGLDIVIIEQEDDSDRHLAQHL	↑Fertile/↓Infertile	0.0000006
3059	P04279	SEM I; Fragm:248–273	HGSKDIFSTQDELLVYNKNQHQTKNL	↑Fertile/↓Infertile	0.001
3083	Q02383	SEM II; Fragm:248–273	HGPKDIFTTQDELLVYNKNQHQTKNL	↑Fertile/↓Infertile	0.010
3938	Q02383	SEM II; Fragm:549–582	ESSESHNIVITEHEVAQDDHLTQQYNEDRNPIST	↑Fertile/↓Infertile	0.001

^1.^ The *m*/*z* values of precursor ions refer to average masses MH^+^. ^2.^ The *p* values were calculated with unpaired *t*-test on peak intensity; significance was set at a *p* value less than 0.05.

## Supplementary Materials

The following supporting information can be downloaded at: https://www.mdpi.com/article/10.3390/ijms231810786/s1.
