# Supplementary material for: Revealing the Hidden Diagnostic Clues of Male Infertility from Human Seminal Plasma by Dispersive Solid Phase Extraction and MALDI-TOF MS
Source: Int J Mol Sci. 2022 Sep 15;23(18):10786. doi: 10.3390/ijms231810786 (PMC9506103; doi:10.3390/ijms231810786)
Supplement: Supplementary file 1 [file ijms-23-10786-s001.zip › ijms-1860479-supplementary.pdf]

Supplementary materials & methods

C_{18} and C_8 *d*-SPE optimization procedure

Adsorption time

Three aliquots (10 mg) of C_{18} and three aliquots (10 mg) of C_8 silica bonded sorbent were added to 100 μL of SP (50 μL of normalized SP sample in 50 μL of deionized water). The slurry was gently stirred at 20 °C for 5, 10 or 15 minutes. The suspensions were then centrifuged at 2000× g for 2 min. The sorbents were then separated from the supernatant and washed twice with 0.1% TFA (20 μL). After the last wash, peptides bound to solid phase were eluted with 15 μL of a 1:1 (v/v) solution of ACN/0.1% TFA. 1 μL of the resulting solution was mixed with 4 μL of 4 mg/mL CHCA solution (50/50 ACN/0.1% TFA) or with 4 μL of saturated solution of SA (35%ACN/0.1% TFA) for MALDI-TOF MS analysis. The total number of peaks were detected over the whole spectra (*m/z* range from 800-20,000 for both CHCA and SA). The results are summarized in Table S1. Best conditions in terms of detected peak number were obtained both at 10 and 15 minutes.

HMS *d*-SPE optimization procedure

Adsorption time

Four aliquots (10 mg) of HMS were mixed with 100 μL of SP (50 μL of normalized SP sample in 50 μL of deionized water) and shaken at room temperature for 5, 10, 15 or 20 minutes. The suspensions were centrifuged at 2000×g for 2 min, then HMS particles were separated from the supernatant and washed twice with 0.1% TFA (20 μL). After the last wash, species retained on HMS were extracted with 25 μL of a 1:1 (v/v) solution of ACN/0.1% TFA and 1 μL of the resulting solution was mixed with 4 μL of 4 mg/mL CHCA solution (50/50 ACN/0.1% TFA) or with 4 μL of saturated solution of SA (35%ACN/0.1% TFA) for MALDI-TOF MS analysis. The total number of peaks were detected

over the whole spectra (*m/z* range from 800-20,000 for both CHCA and SA). The results are summarized in Table S1. Best conditions in terms of detected peak number were obtained both at 15 and 20 minutes.

Table S1. Total number of detected peaks *vs* adsorption times.

**Solid phase type**	**Mean[a] peaks number S/N ≥10**							
	**SA**				**CHCA**			
	**A_t [b] 5 min**	**A_t 10 min**	**A_t 15 min**	**A_t 20 min**	**A_t 5 min**	**A_t 10 min**	**A_t 15 min**	**A_t 20 min**
**C_{18}**	85 ± 1 [c]	126 ± 6	120 ± 5	NA	82 ± 4	99 ± 5	95 ± 4	NA
**C_8**	77 ± 3	98 ± 2	90 ± 3	NA	41 ± 1	55 ± 3	52 ± 2	NA
**HMS**	67 ± 2	77±4	95 ± 3	90 ± 3	26 ± 2	29 ± 1	37 ± 2	35 ± 2

[a] Mean peaks number was calculated on three replicate spectra. Spectra were acquired in an *m/z* range from 800 to 20,000 for both SA and CHCA matrix. [b] A_t, Adsorption time. [c] Values are expressed as mean ± standard deviation (SD).

C_{18} and C_8 *d*-SPE optimization procedure

Washing step

Two aliquots (10 mg) of C_{18} and two aliquots (10 mg) of C_8 silica bonded sorbent were added to 100 µL of SP (50 µL of normalized SP sample in 50 µL of deionized water). The slurry was gently stirred at 20 °C for 10 minutes. The suspensions were then centrifuged at 2000×g for 2 min and the sorbents were then separated from the supernatant. One aliquot of each type of sorbent was washed twice with 0.1% TFA (20 µL) while the other aliquot was washed three times with the same washing solution. After the last wash, peptides bound to solid phase were eluted with 15 µL of a 1:1 (v/v) solution of

ACN/0.1% TFA. 1 μL of the resulting solution was mixed with 4 μL of 4 mg/mL CHCA solution (50/50 ACN/0.1% TFA) or with 4 μL of saturated solution of SA (35%ACN/0.1% TFA) for MALDI-TOF MS analysis. The total number of peaks were detected over the whole spectra (*m/z* range from 800-20,000 for both CHCA and SA). The results are summarized in Table S2. Best conditions in terms of detected peak number were obtained for two washing steps.

HMS *d*-SPE optimization procedure

Washing step

Two aliquots (10 mg) of HMS were mixed with 100 μL of SP (50 μL of normalized SP sample in 50 μL of deionized water) and shaken at room temperature for 15 minutes. The suspensions were centrifuged at 2000×g for 2 min, then HMS particles were separated from the supernatant. One aliquot was washed twice with 0.1% TFA (20 μL) while the other aliquot was washed three times with the same washing solution. After the last wash, species retained on HMS were extracted with 25 μL of a 1:1 (v/v) solution of ACN/0.1% TFA and 1 μL of the resulting solution was mixed with 4 μL of 4 mg/mL CHCA solution (50/50 ACN/0.1% TFA) or with 4 μL of saturated solution of SA (35%ACN/0.1% TFA) for MALDI-TOF MS analysis. The total number of peaks was detected over the whole spectra (*m/z* range from 800-20,000 for both CHCA and SA). The results are summarized in Table S2. Best conditions in terms of detected peak number were obtained for two washing steps.

Table S2. Total number of detected peaks *vs* washing steps.

Solid phase type	Mean [a] peaks number S/N ≥ 10			
	SA		CHCA	
	2 washing steps	3 washing steps	2 washing steps	3 washing steps
C_{18}	128 ± 4 [b]	86 ± 2	109 ± 5	97 ± 2
C_8	103 ± 1	67 ± 3	60 ± 4	53 ± 3
HMS	101 ± 3	70 ± 2	40 ± 1	34 ± 2

[a] Mean peaks number was calculated on three replicate spectra. Spectra were acquired in an *m/z* range from 800 to 20,000 for both SA and CHCA matrix. [b] Values are expressed as mean ± standard deviation (SD).

C_{18} and C_8 *d*-SPE optimization procedure

Elution step

Three aliquots (10 mg) of C_{18} and three aliquots (10 mg) of C_8 silica bonded sorbent were added to 100 µL of SP (50 µL of normalized SP sample in 50 µL of deionized water). The slurry was gently stirred at 20 °C for 10 minutes. The suspensions were then centrifuged at 2000×g for 2 min and the sorbents were then separated from the supernatant and washed twice with 0.1% TFA (20 µL). After the last wash, peptides bound to solid phase were eluted with 10, 15 or 25 µL of a 1:1 (v/v) solution of ACN/0.1% TFA. 1 µL of the resulting solution was mixed with 4 µL of 4 mg/mL CHCA solution (50/50 ACN/0.1% TFA) or with 4 µL of saturated solution of SA (35%ACN/0.1% TFA) for MALDI-TOF MS analysis. The total number of peaks were detected over the whole spectra (*m/z* range from 800-20,000 for both CHCA and SA). The results are summarized in Table S3. Best conditions in terms of detected peak number were obtained for 15 µL of elution solution.

HMS *d*-SPE optimization procedure

Elution step

Four aliquots (10 mg) of HMS were mixed with 100 µL of SP (50 µL of normalized SP sample in 50 µL of deionized water) and shaken at room temperature for 15 minutes. The suspensions were centrifuged at 2000×g for 2 min, then HMS particles were separated from the supernatant and washed twice with 0.1% TFA (20 µL). After the last wash, species retained on HMS were extracted with 10, 15, 25 or 30 µL of a 1:1 (v/v) solution of ACN/0.1% TFA and 1 µL of the resulting solution was mixed with 4 µL of 4 mg/mL CHCA solution (50/50 ACN/0.1% TFA) or with 4 µL of saturated solution of SA (35%ACN/0.1% TFA) for MALDI-TOF MS analysis. The total number of peaks was detected over the whole spectra (*m/z* range from 800-20,000 for both CHCA and SA). The results are summarized in Table S3. Best conditions in terms of detected peak number were obtained for 25 µL of elution solution.

Table S3. Total number of detected peaks *vs* elution volumes.

**Solid phase type**	**Mean[a] peaks number S/N ≥ 10**							
	**SA**				**CHCA**			
	**Eluent 10 µl**	**Eluent 15 µl**	**Eluent 25 µl**	**Eluent 30 µl**	**Eluent 10 µl**	**Eluent 15 µl**	**Eluent 25 µl**	**Eluent 30 µl**
**C_{18}**	90 ± 2 [b]	141 ± 5	110 ± 6	NA	88 ± 3	124 ± 5	108 ± 4	NA
**C_8**	85 ± 1	109 ± 3	93 ± 4	NA	66 ± 2	73 ± 1	68 ± 3	NA
**HMS**	80 ± 4	95 ± 1	110 ± 2	104 ± 3	35 ± 4	39 ± 2	50 ± 1	46 ± 2

[a] Mean peaks number was calculated on three replicate spectra. Spectra were acquired in an *m/z* range from 800 to 20,000 for both SA and CHCA matrix. [b] Values are expressed as mean ± standard deviation (SD).

Optimization of MALDI-TOF sample preparation in CHCA for the different *d*-SPE sorbents.

Three CHCA matrix solutions were prepared at the concentration of 4 mg/ml, one dissolving CHCA in a solution of 50% ACN in 0.1%TFA, one dissolving CHCA in a solution of 70% ACN in 0.1%TFA and one dissolving CHCA with ACN, 0.1% TFA and 25mM ammonium bicarbonate, in a ratio of 60:20:20 (Table S4). The obtained solutions were then sonicated for 1 min. 1 µL of SP previously treated with HMS, C_{18} or C_8 sorbent, was mixed with 4 µL of each CHCA preparation. Then 1 µL of the resulting mixtures was spotted on the MALDI target plate (Opti-TOF 384-Well Insert, ABSciex,Framingham, MA, USA). Six replicate spectra were acquired in the *m/z* range between 800 and 20,000 with $S/N \geq 10$. Mean Percentage Coefficient of variation (CV%) was then calculated for peak height, peak area and for S/N (Table S4).

Optimization of MALDI-TOF sample preparation in SA for the different *d*-SPE sorbents.

Three SA matrix solutions were obtained, one preparing a saturated solution of SA in 50% ACN/0.1%TFA, the other preparing a saturated solution of SA in 35% ACN/0.1%TFA and last preparing a saturated solution of SA in 35% 0.1%TFA/ACN (Table S4). The obtained solutions were then sonicated for 1 min. 1 µL of SP previously treated with HMS, C_{18} or C_8 sorbent, was mixed with 4 µL of each SA preparation. Then 1 µL of the resulting mixtures was spotted on the MALDI target plate (Opti-TOF 384-Well Insert, ABSciex,Framingham, MA, USA). Six replicate spectra were acquired in the *m/z* range between 800 and 20,000 with $S/N \geq 10$. Mean CV% was then calculated for peak height, peak area and for S/N (Table S4).

Table S4. Mean peak number and CVs on peak height, area and S/N for different MALDI sample preparations in CHCA and SA.

Matrix	MALDI Sample Preparation[a]	C_{18}				C_8				HMS			
		**Mean Peak Number[b]**	**CV (%) Height[c]**	**CV (%) Area[d]**	**CV (%) S/N[e]**	**Mean Peaks Number**	**CV (%) Height**	**CV (%) Area**	**CV (%) S/N**	**Mean Peaks Number**	**CV (%) Height**	**CV (%) Area**	**CV (%) S/N**
CHCA[f]	50%ACN in 0.1%TFA	83 ± 2	9.4 ± 1	8.2 ± 1	7.1 ± 2	76 ± 3	10.7 ± 3	7.6 ± 1	7.7 ± 1	71 ± 2	11.7 ± 2	9.2 ± 1	9.3 ± 1
	70%ACN in 0.1%TFA	79 ± 2	10 ± 2	9.6 ± 3	8.8 ± 1	64 ± 1	12.6 ± 1	12.9 ± 2	10.4 ± 3	49 ± 1	13.6 ± 3	12.2 ± 1	11.7 ± 3
	ACN, 0.1% TFA and 25mM AMBIC[g] (60:20:20)	80 ± 3	9.2 ± 3	7.5 ± 1	7.4 ± 2	67 ± 2	11.2 ± 2	11.9 ± 2	9 ± 1	54 ± 1	12.5 ± 2	11.7 ± 2	10.1 ± 2
SA	Saturated solution in 50% ACN/0.1%TFA	133 ± 4	8.8 ± 1	10 ± 3	8.2 ± 2	109 ± 5	9 ± 1	11.7 ± 3	12.9 ± 1	110 ± 3	12 ± 1	11.8 ± 1	10.8 ± 3
	Saturated solution in 35% ACN/0.1%TFA	140 ± 5	8.4 ± 2	9.6 ± 2	7.9 ± 2	113 ± 4	8.8 ± 2	11.3 ± 2	12.6 ± 2	114 ± 2	11.6 ± 3	11.4 ± 1	10.3 ± 2
	Saturated solution in 35% 0.1%TFA / ACN	126 ± 2	9.1 ± 3	10.6 ± 1	8.6 ± 1	100 ± 2	9.6 ± 1	12.3 ± 4	13 ± 3	98 ± 4	12.5 ± 2	12 ± 2	11.4 ± 1

[a] Samples were spotted on MALDI-target plate adopting *dried-droplet* method. Sample-to matrix ratio was 1:4. [b] Mean peaks number from six replicate spectra was calculated. The spectra were acquired in the *m/z* range between 800 and 20,000 for both SA and CHCA, with S/N ≥ 10. [c] Mean Percentage Coefficient of variation (CV%) calculated for height. [d] Mean Percentage Coefficient of variation (CV%) calculated for peak area. [e] Mean Percentage Coefficient of variation (CV%) calculated for S/N. [f] CHCA matrix solution was prepared in a concentration of 4 mg/ml in all the *d*-SPE preparations. [g] AMBIC, Ammonium Bicarbonate.

Table S5. Total protein content of SP specimen from each study participant.

Sample ID	Study groups	Total protein concentration (mg/ml)[a]	Mean[b] total protein concentration
1	Fertile	50.0	54.4 ± 9.8
2	Fertile	57.6	
3	Fertile	51.8	
4	Fertile	47.6	
5	Fertile	72.0	
6	Fertile	76.7	
7	Fertile	57.1	
8	Fertile	49.8	
9	Fertile	47.7	
10	Fertile	38.8	
11	Fertile	50.4	
12	Fertile	49.6	
13	Fertile	63.4	
14	Fertile	53.6	
15	Fertile	49.9	
1	Infertile	43.6	50.2 ± 14.4
2	Infertile	52.9	
3	Infertile	35.5	
4	Infertile	82.0	
5	Infertile	56.5	
6	Infertile	72.7	
7	Infertile	33.8	
8	Infertile	30.7	
9	Infertile	38.0	
10	Infertile	56.7	
11	Infertile	42.1	
12	Infertile	52.2	
13	Infertile	54.8	
14	Infertile	41.6	
15	Infertile	59.2	

[a] Total protein concentration (mg/ml) of SP samples was determined by the bicinchoninic acid (BCA) assay.
[b] Values are expressed as mean ± standard deviation (SD).

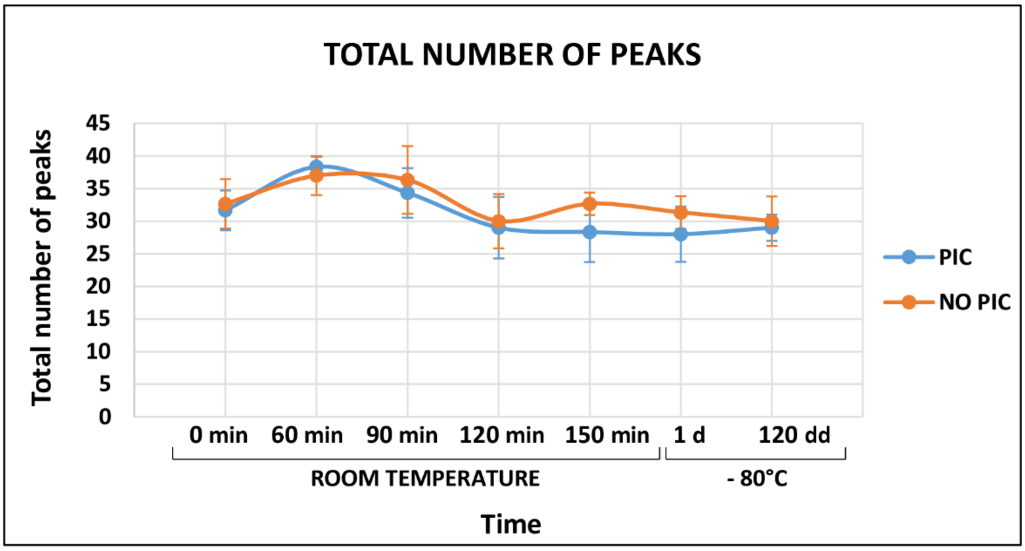

Figure S1. Assessment of protease activity in SP. Total mean number of peaks detected from a SP sample of a normozoospermic donor analyzed with and without the use of PIC. The sample was prepared in three independent experiments and the peak number was calculated immediately (time= 0) and after 60, 90, 120 and 150 min at room temperature. Then the samples were frozen at -80°C and the total peaks number was analyzed after 1 day and after 120 days. The mean peak number and the standard deviation were calculated on three replicate spectra in the 800-20,000 *m/z* range using SA matrix (S/N≥10).

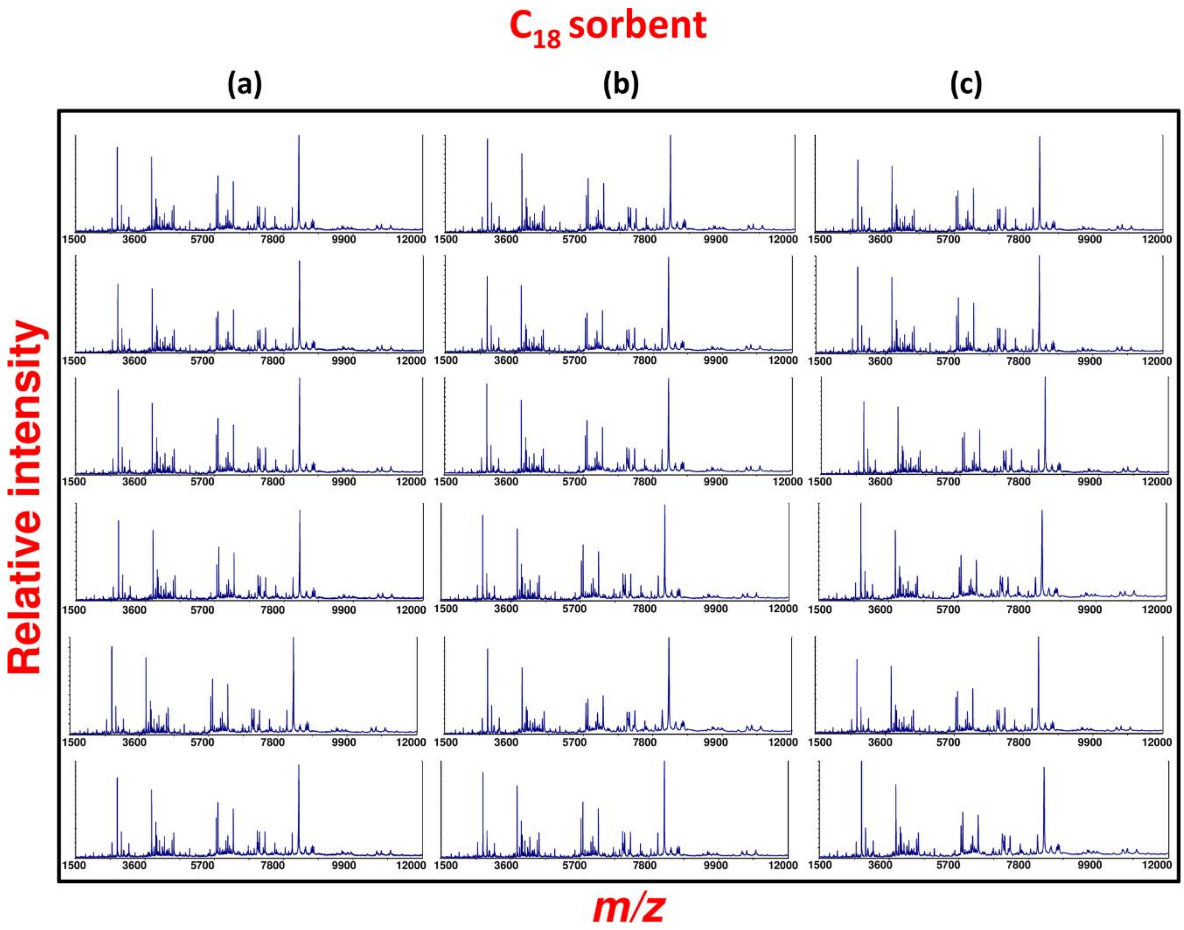

Figure S2. Repeatability of MALDI-TOF SP peptidic profiles after C_{18} sorbent enrichment. The SP sample from a normozoospermic donor was processed by C_{18} sorbent in three independent experiments (a,b,c); for each experiment, the sample was run in six replicates, thus for each preparation six spectra were acquired, resulting in a total of 18 MALDI mass spectra. The MALDI mass spectra were recorded in linear mode using SA as matrix.

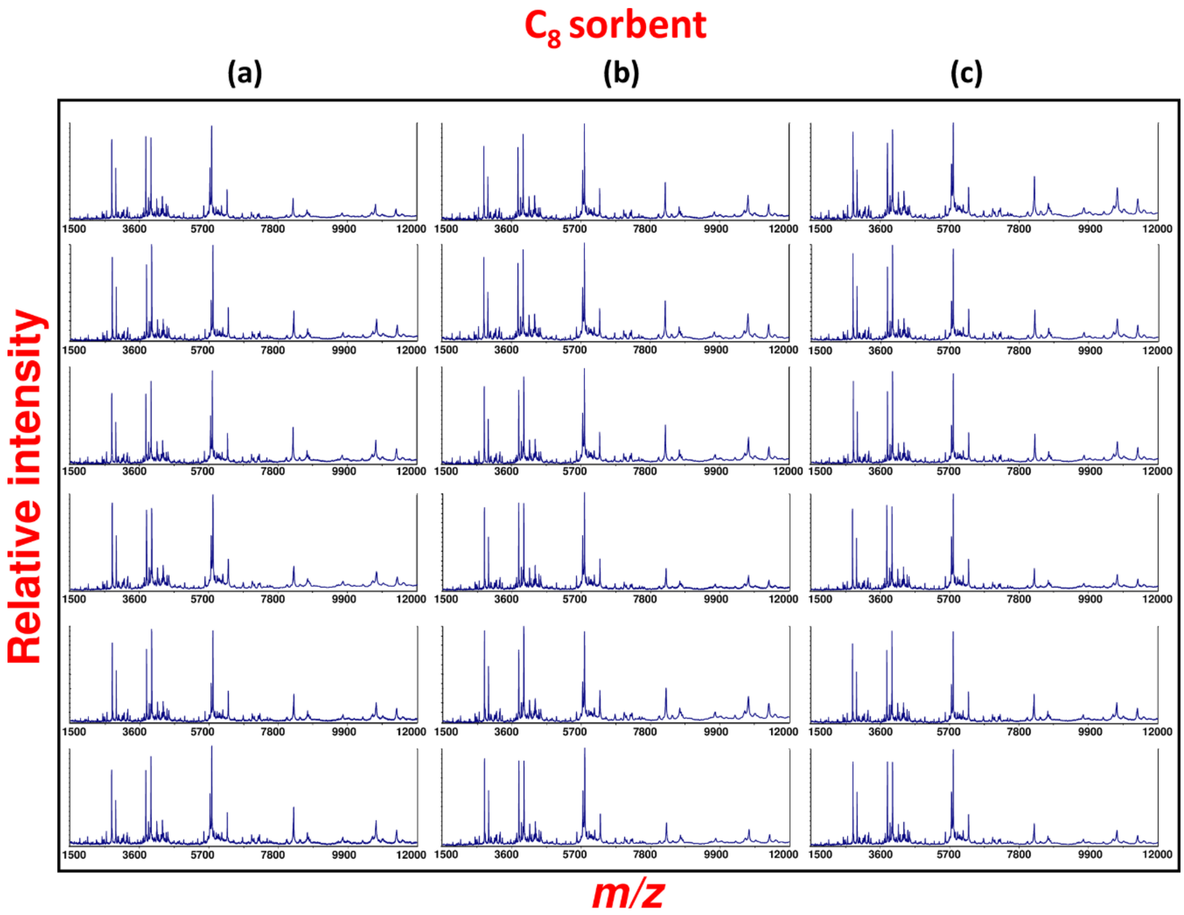

Figure S3. Repeatability of MALDI-TOF SP peptidic profiles after C_8 sorbent enrichment. The SP sample from a normozoospermic donor was processed by C_8 sorbent in three independent experiments (a,b,c); for each experiment, the sample was run in six replicates, thus for each preparation six spectra were acquired, resulting in a total of 18 MALDI mass spectra. The MALDI mass spectra were recorded in linear mode using SA as matrix.

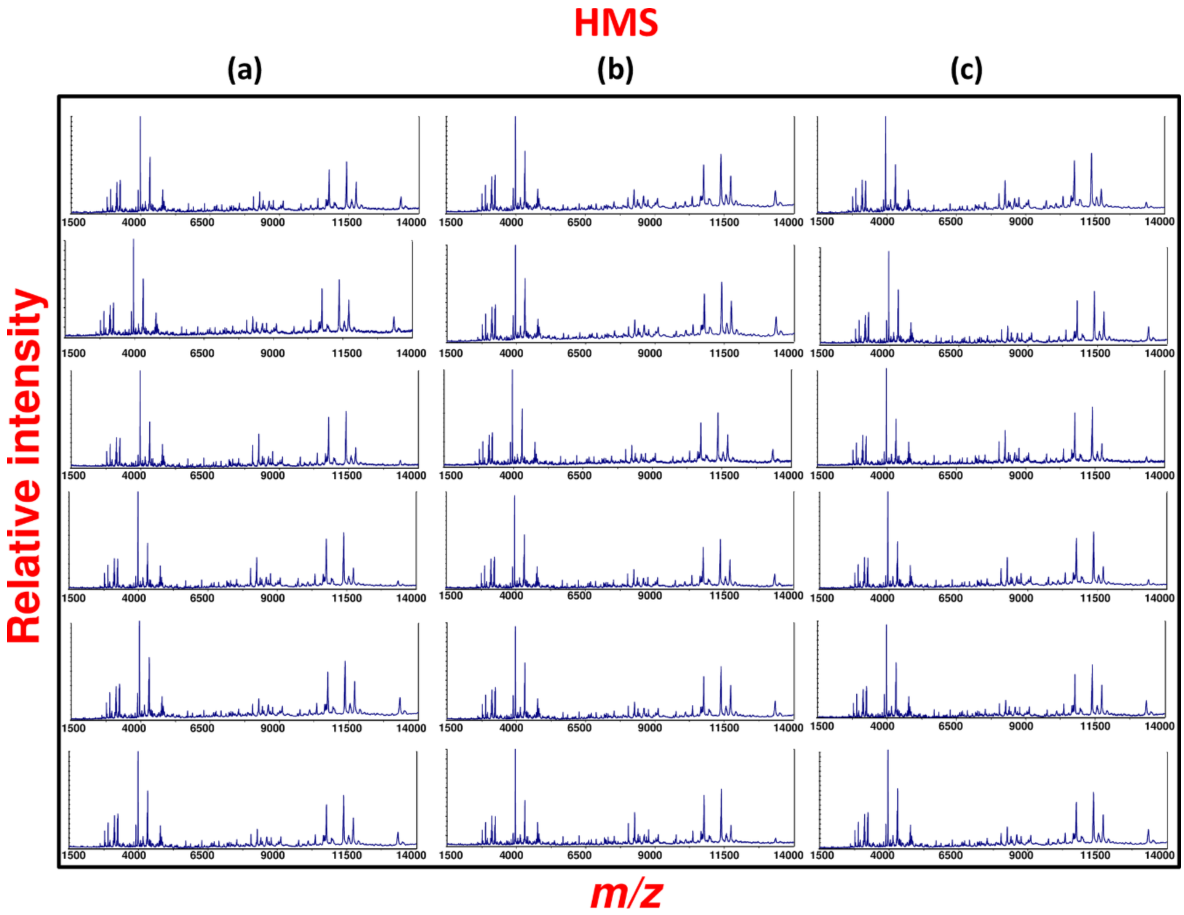

Figure S4. Repeatability of MALDI-TOF SP peptidic profiles after HMS sorbent enrichment. The SP sample from a normozoospermic donor was processed by HMS in three independent experiments (a,b,c); for each experiment, the sample was run in six replicates, thus for each preparation six spectra were acquired, resulting in a total of 18 MALDI mass spectra. The MALDI mass spectra were recorded in linear mode using SA as matrix.

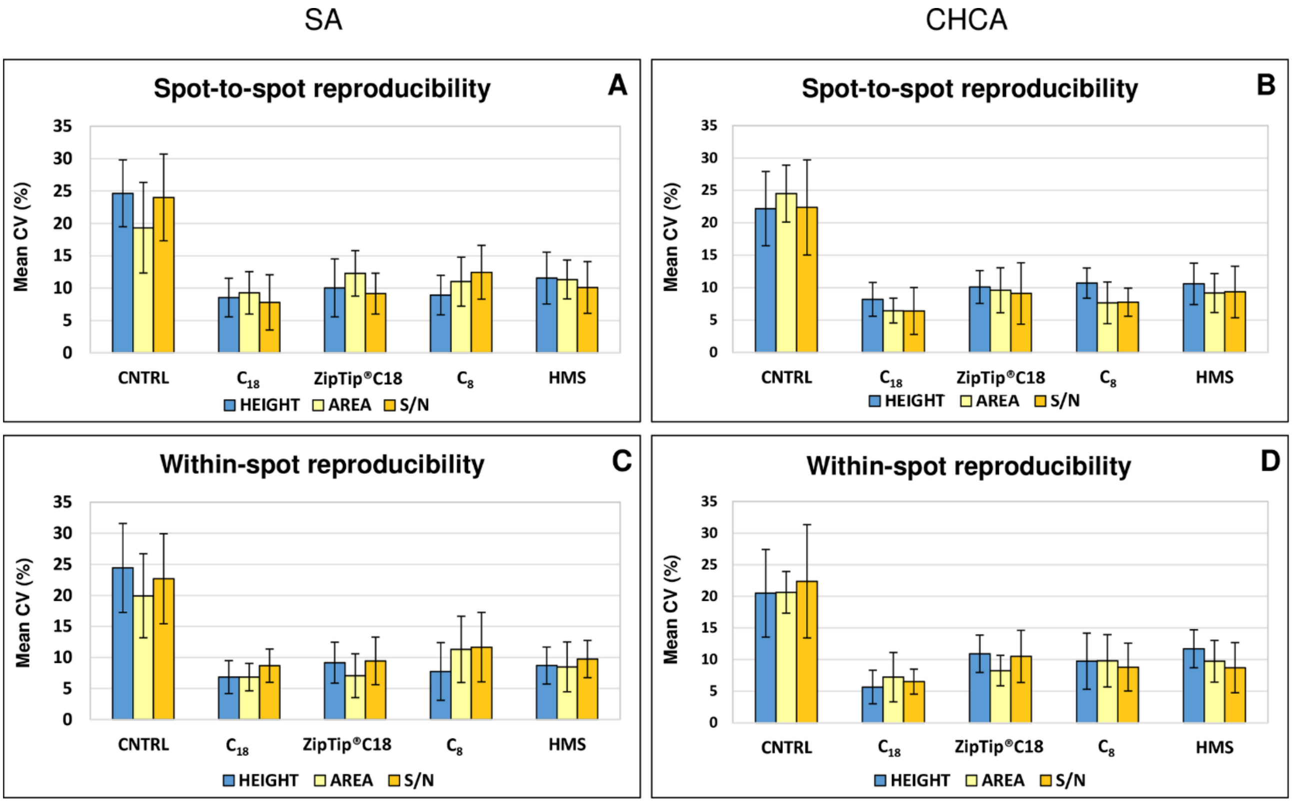

Figure S5. Spot-to-spot and within-spot reproducibility of the method. Spot-to-spot and within-spot reproducibility assessment for peak height, peak area and S/N in acquired MALDI-TOF mass spectra of SP from the same subject before and after processing by C_{18} sorbent, ZipTip®C18, C_8 sorbent and HMS, using SA (Panels A and C) and CHCA (Panels B and D) matrix solutions. The mean CV (%) and the standard deviations were calculated from 30 selected peaks in three independent experiments. The mean CV (%) was calculated from the ratio between SD and the mean of peak height, peak area, and S/N.

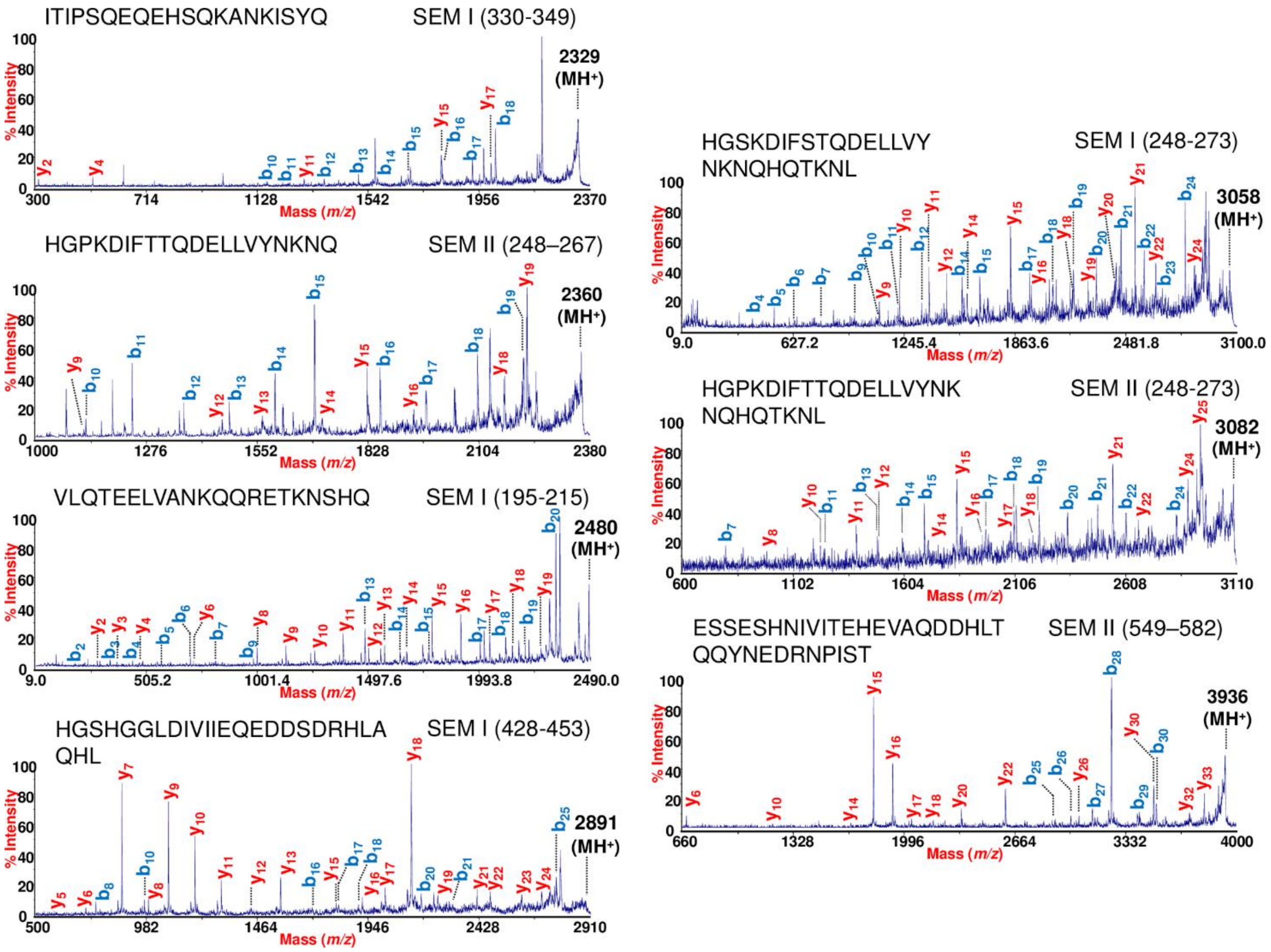

Figure S6. MALDI-TOF/TOF mass spectra of peptides resulted significantly different between fertile and infertile men. MALDI-TOF/TOF mass spectra with fragment ion signals obtained by direct sequencing of Semenogelin I and Semenogelin II derived peptides: ITIPSQEQEHSQKANKISYQ (SEM I, residues 330 to 349), HGPKDIFTTQDELLVYNKNQ (SEM II, residues 248 to 267), VLQTEELVANKQQRETKNSHQ (SEM I, residues 195 to 215), HGSHGGLDIVIIEQEDDSDRHLAQHL (SEM I, residues 428 to 453), HGSKDIFSTQDELLVYNKNQHQTKNL (SEM I, residues 248 to 273), HGPKDIFTTQDELLVYNKNQHQTKNL (SEM II, residues 248 to 273), ESSESHNIVITEHEVAQDDHLTQQYNEDRNPIST (SEM II, residues 549 to 582). The *m/z* values of precursor ions refer to monoisotopic masses MH^+.
